# Reduced Ca^2+^ transient amplitudes may signify increased or decreased depolarization depending on the neuromodulatory signaling pathway

**DOI:** 10.3389/fnins.2022.931328

**Published:** 2022-07-22

**Authors:** Arunima Debnath, Paul D. E. Williams, Bruce A. Bamber

**Affiliations:** ^1^Department of Biological Sciences, The University of Toledo, Toledo, OH, United States; ^2^Department of Biomedical Sciences, College of Veterinary Medicine, Iowa State University, Ames, IA, United States

**Keywords:** neuromodulation, Ca^2+^ dynamics, depolarization, signal integration, graded potentials

## Abstract

Neuromodulators regulate neuronal excitability and bias neural circuit outputs. Optical recording of neuronal Ca^2+^ transients is a powerful approach to study the impact of neuromodulators on neural circuit dynamics. We are investigating the polymodal nociceptor ASH in *Caenorhabditis elegans* to better understand the relationship between neuronal excitability and optically recorded Ca^2+^ transients. ASHs depolarize in response to the aversive olfactory stimulus 1-octanol (1-oct) with a concomitant rise in somal Ca^2+^, stimulating an aversive locomotory response. Serotonin (5-HT) potentiates 1-oct avoidance through Gα_q_ signaling, which inhibits L-type voltage-gated Ca^2+^ channels in ASH. Although Ca^2+^ signals in the ASH soma decrease, depolarization amplitudes increase because Ca^2+^ mediates inhibitory feedback control of membrane potential in this context. Here, we investigate octopamine (OA) signaling in ASH to assess whether this negative correlation between somal Ca^2+^ and depolarization amplitudes is a general phenomenon, or characteristic of certain neuromodulatory pathways. Like 5-HT, OA reduces somal Ca^2+^ transient amplitudes in ASH neurons. However, OA antagonizes 5-HT modulation of 1-oct avoidance behavior, suggesting that OA may signal through a different pathway. We further show that the pathway for OA diminution of ASH somal Ca^2+^ consists of the OCTR-1 receptor, the G_o_ heterotrimeric G-protein, and the G-protein activated inwardly rectifying channels IRK-2 and IRK-3, and this pathway reduces depolarization amplitudes in parallel with somal Ca^2+^ transient amplitudes. Therefore, even within a single neuron, somal Ca^2+^ signal reduction may indicate either increased or decreased depolarization amplitude, depending on which neuromodulatory signaling pathways are activated, underscoring the need for careful interpretation of Ca^2+^ imaging data in neuromodulatory studies.

## Introduction

Neuromodulation alters neuronal circuit function through regulation of neuronal excitability and synaptic strengths, thereby reconfiguring circuit outputs (Bargmann, 2012; Marder, 2012). Well studied neuromodulators include monoamines such as serotonin (5-HT) and dopamine, and neuropeptides such as vasopressin and oxytocin. Neuromodulators function through G-protein signaling cascades to regulate behavior in both vertebrates and invertebrates, including feeding behavior, learning and memory, attention and arousal (Hasselmo, 1999, 2006; Lee et al., 2004; Sara, 2009; Al-Anzi et al., 2010; Harris-Warrick and Johnson, 2010; Elphick, 2012; Sara and Bouret, 2012; Ko et al., 2015; Schwarz and Luo, 2015; Ballinger et al., 2016; Koelle, 2018; Nässel et al., 2019; Bacqué-Cazenave et al., 2020; Ranjbar-Slamloo and Fazlali, 2020). Multiple different neuromodulators often act on the same neuron through different receptors and signaling pathways resulting in differential behavioral effects (Chase and Koelle, 2007; Wragg et al., 2007; Li et al., 2018). G-protein coupled receptors (GPCRs) involved in neuromodulation signal through the heterotrimeric G-protein families G_s_, G_q_, and G_i/o_, which dissociate into activated Gα subunits and Gβγ subunits upon activation by GPCRs. G_s_ signaling increases neurotransmission through the adenylyl cyclase/protein kinase A pathway (Reynolds et al., 2005; Schade et al., 2005; Koelle, 2018). G_q_ signaling increases neurotransmission through the downstream effectors phospholipase Cβ and the Rho guanine nucleotide exchange factor Trio. Phospholipase Cβ generates diacylglycerol (DAG) and inositol triphosphate (IP_3_), which gates Ca^2+^ release from intracellular stores, while Trio also promotes increased DAG levels. DAG and Ca^2+^ increase synaptic vesicle and dense core vesicle release through UNC-13 and protein kinase C (Koelle, 2018), while Ca^2+^ plays other, independent, roles in cell signaling (see below). G_i/o_ signaling inhibits neurotransmission through several potential pathways including inhibition of adenylyl cyclase and antagonism of Gα_q_ signaling by Gα_i/o_ (Koelle, 2018; Muntean et al., 2021). However, the predominant mechanism of G_i/o_ signaling appears to be through the release of Gβγ subunits upon GPCR activation, which hyperpolarize membranes by activating G-protein coupled inward rectifying potassium channels (GIRKs) (Kano et al., 2019), inhibit adenylate cyclase (Muntean et al., 2021), reduce synaptic vesicle release by inhibiting voltage-gated Ca^2+^ channels (VGCCs) at presynaptic terminals (Waterman, 2000), and directly inhibit synaptic vesicle release by binding key components of the synaptic vesicle release machinery (Blackmer et al., 2001; Hamid et al., 2014; Hamm and Alford, 2020; Alten et al., 2022).

The study of neuromodulation in the context of intact circuits is greatly facilitated by non-invasive imaging techniques. Ca^2+^ imaging is a powerful and widely used approach (Patriarchi et al., 2018; Corkrum et al., 2020; Redolfi et al., 2021) and, thus far, preferred over fluorescent voltage indicators due to superior sensitivity, kinetics, and ease of expression (Vogt, 2015; Inagaki and Nagai, 2016; Mollinedo-Gajate et al., 2019; Inoue, 2021; Zhu et al., 2021). Ca^2+^ imaging utilizes fluorescent calcium indicators, often encoded by transgenes with neuron specific promoters, enabling selective labeling in nervous system areas with high cellular heterogeneity. Quantitatively, Ca^2+^ signal amplitudes and depolarization amplitudes are believed to rise or fall in parallel (Akerboom et al., 2012; Shidara et al., 2013; Kato et al., 2014; Ghosh et al., 2016). However, examples have been documented where this positive correlation does not hold true (Ferkey et al., 2007; Lindy et al., 2014; Zahratka et al., 2015). Ca^2+^ signals are an indirect representation of neuronal depolarization, since Ca^2+^ enters the cytoplasm through VGCCs, which activate downstream of depolarization. Potential sources of error include non-linearity in the ratio of Ca^2+^ to depolarization introduced by the modulation of VGCCs by intracellular signaling pathways and the release of Ca^2+^ from internal stores, which creates a Ca^2+^ pool independent of depolarization. Furthermore, Ca^2+^ itself is a potent second messenger which can modulate membrane potential by activating downstream ion channels (Grissmer, 2007). For these reasons, interpretation of a change in Ca^2+^ transient amplitude is not always straightforward.

Recent studies of neuromodulation in the nematode *Caenorhabditis elegans* have provided a particularly salient example of the need to carefully interpret changes in Ca^2+^ transient amplitudes. The aversive response of *C. elegans* to 1-octanol (1-oct) is mediated by ASH polymodal nociceptive neurons and is heavily modulated by monoamines and neuropeptides (Chao et al., 2004; Wragg et al., 2007; Harris et al., 2010; Komuniecki et al., 2012; Mills et al., 2012). 5-HT potentiates aversive locomotory responses to sub-maximal 1-oct stimulation (i.e., 1-oct diluted to 30% in ethanol), in part, through the Gα_q_-coupled SER-5 receptor in ASHs (Harris et al., 2009). Surprisingly, ASH somal Ca^2+^ transients are reduced in this context (Zahratka et al., 2015). However, 5-HT actually increases depolarization amplitudes because SER-5/Gα_q_ reduces Ca^2+^ influx, and Ca^2+^ inhibits ASHs through Ca^2+^ activated K^+^ channels (K_Ca_) (Zahratka et al., 2015; Williams et al., 2018b). Is this inverse relationship between somal Ca^2+^ transient amplitude and depolarization amplitude a general characteristic of this particular neuron, or is it necessary to evaluate each instance of modulation individually, even within the same neuron type? To answer this question, we have compared the modulation of ASHs by the neuromodulator octopamine (OA), which diminishes 1-oct responses through the OCTR-1 receptor expressed in ASHs.

## Materials and methods

### Strains and constructs

Strains were cultured on nematode growth media (NGM) plates seeded with *Escherichia coli* OP50 bacteria as per standard protocols. The following strains were used: N2 (Bristol wild type), FY928 *grIs17* [*Psra-6::GCaMP3*] (wild type expressing GCaMP3 in ASH), VC224 *octr-1(ok371)* (*octr-1* null mutants), FY991 *octr-1(ok371); grIs17* [*Psra-6::GCaMP3*] (*octr-1* null mutants expressing GCaMP3 in ASH), FY1030 *ser-3(ad1774); grIs17* [*Psra-6::GCaMP3*] (*ser-3* null mutants expressing GCaMP3 in ASH), FY1053 *irk-1(n4895); grIs17* [*Psra-6::GCaMP3*] (*irk-1* null mutants expressing GCaMP3 in ASH), FY1056 *irk-2(n4896); grIs17* [*Psra-6::GCaMP3*] (*irk-2* null mutants expressing GCaMP3 in ASH), FY1061 *irk-3(n5049); grIs17* [*Psra-6::GCaMP3*] (*irk-3* null mutants expressing GCaMP3 in ASH), FY1063 *goa-1(n1134); grIs17* [*Psra-6::GCaMP3*] (*goa-1* null mutants expressing GCaMP3 in ASH), MT15934 *irk-1(n4895)* (*irk-1* null mutants), FQ295 *irk-2(n4896)* (*irk-2* null mutants), MT17360 *irk-3(n5049)* (*irk-3* null mutants), and LX1751 *lin-15(n765)* X; *vsEx700* (ASH::PTX worms, expressing pertussis toxin (PTX) in ASH under the control of the *osm-10* promoter). The P*sra-6*::GCaMP3 transgene was obtained as the extrachromosomal array *kyEx2865* in strain CX10979 (Kato et al., 2014). The P*osm-10*::PTX transgene was obtained as extrachromosomal array *vsEx700* in strain LX1751 (Hofler and Koelle, 2011). MT15934, FQ295, and MT17360 were kindly supplied by N. Ringstad (Skirball Institute, New York, NY, United States), LX1751 was a kind gift from M. Koelle (Yale University, New Haven, CT, United States), and CX10979 was generously supplied by C. Bargmann.

### Calcium imaging

Calcium imaging experiments were performed as previously described (Mills et al., 2012; Zahratka et al., 2015; Williams et al., 2018b). Worms were glued down using WormGlu cyanoacrylate glue (GluStitch, Delta, BC, Canada), on a 15 mm diameter round coverslip coated with Sylgard (Dow Corning, Midland, MI, United States), immersed in external solution (see below). The coverslips were then placed in a laminar flow chamber (Warner RC26G, Warner Instruments, Hamden, CT, United States) which was continuously perfused with external solution; 150 mM NaCl, 5 mM KCl, 5 mM CaCl_2_, 1 mM MgCl_2_, 10 mM glucose, 15 mM HEPES, pH 7.30, 327–333 mOsm. Saturated 1-oct (2.37 mM in external solution) was delivered under gravity feed through Luer valves using a perfusion pencil (AutoMate Scientific, Berkeley, CA, United States) or homemade equivalent. Fluorescent tracer sulforhodamine 101 (SR101, 1 μM, Thermo Fisher Scientific) was used in the 1-oct solution for visual inspection of flow and to check for staining of the worm’s nose, indicative of successful stimulus application. All solutions were delivered using the perfusion pencil as described above, mounted on a SF77B Perfusion Fast Step device (step size 200 μM, Warner Instruments, Hamden, CT, United States) to provide precise computer control of pipette position. After each exposure, we visually examined the animals for SR101 staining. No response was observed in ASHs to external solution containing 1 μM SR101 alone. The *sra-6* promoter is active in ASH and ASI neurons, which are distinguishable based on their relative positions in the head. ASI neurons did not respond to 1-oct.

We partially dissected the animals to expose the ASH to external solution for Ca^2+^ imaging experiments with high K^+^ stimulation and in experiments where OA was acutely applied to the ASHs. Dissection was performed according to previously described procedure (Goodman et al., 1998). The ASH was exposed by slitting the cuticle with a glass patch pipette (TW150-3, World Precision Instruments) that had been melted, drawn to a fine point and broken back to create a sharp-ended cutter using a Narishige MF-83 microforge (Narishige, Setagaya-ku, Tokyo, Japan). Cutters were mounted on a micromanipulator (Sutter MP285, Sutter Instruments, Novato, CA, United States) to slit open the cuticle, exposing one of the two ASHs to the external bath solution (Williams et al., 2018b). High K^+^ external solution was composed of 120 mM NaCl, 30 mM KCl, 1 mM MgCl_2_, 15 mM HEPES, 5 mM CaCl_2_, 10 mM glucose, with pH 7.3, 327–333 mOsm. High K^+^ containing external solution was applied using a four-barrel glass puffer with barrel cross section of 300 μm, mounted on a SF77B Perfusion Fast Step device. Solutions were delivered using a syringe pump (KD Scientific, Holliston, MA, United States). Recordings were performed on an Axioskop 2 FS Plus upright compound microscope (40× Achroplan water immersion objective, GFP filter set # 38) (Zeiss, Germany), fitted with an Orca ER CCD camera (Hamamatsu, Skokie, IL, United States) and an automated shutter (Uniblitz, Vincent Associates, Rochester, NY, United States). Minimal illumination intensity was used to prevent GCaMP3 photobleaching, and we did not observe differential photobleaching rates between different genotypes and treatment groups. Exposure times were 50 ms with 4× binning and frame rate of 15 frames/s. Monoamine (OA/5-HT/OA + 5-HT, Sigma-Aldrich, St. Louis, MO, United States) exposure was performed by either incubating the animals on NGM plates containing monoamine(s) at 4 mM each, or by direct application to ASH neurons in partially dissected worms. For plate incubation, each monoamine was added to the NGM agar at 4 mM, and worms were incubated for 30–45 min. OA, 5-HT, OA + 5-HT plates were prepared fresh on each day of recording. Direct application of OA was achieved using a two-opening theta (θ) glass tube that had been heated and drawn to a fine caliber by hand. A PE10 polyethylene tube (0.61 OD × 0.28 ID) (Warner Instruments, Hamden, CT, United States) was inserted into the back of each barrel and sealed using Sylgard to supply external (control) and OA-containing solutions. Solutions flow was regulated using nylon three-way Luer Lock stopcocks; and delivered, using a syringe pump (KD Scientific, Holliston, MA, United States), such that only one barrel of the theta tubing (i.e., buffer or OA) was flowing at a time. Either stream delivered from the dual-chambered pipette was able to shield the exposed ASH from the 1-oct stimulus; inadvertent exposure of exposed ASHs was immediately obvious due to SR101 staining, and such recordings were discarded. Fluorescent images were acquired *via* MetaVue 7.6.5 (MDS Analytical Technologies, Sunnyvale, CA, United States). For quantifying fluorescence changes in the soma, a square region of interest was drawn, such that the entire soma was the only fluorescent object present. For ASH amphids, a square region was drawn around the ciliated endings located at the worm’s nose, excluding the dendrite. The acquired images were analyzed using Jmalyze software (Rex Kerr). Roughly circular or oval regions of interest limited to the cell soma or amphid were tracked using Jmalyze. Fluorescence amplitude was calculated as ΔF/F_o_, where the fluorescence at a given time point (F) minus the fluorescence immediately before stimulus application (F_o_) was divided by F_o_ [i.e., (F − F_o_)/F_o_]. For quantitative comparisons, the maximal ΔF/F_o_ over the time course of the stimulus was plotted in the bar graphs and used for statistical analysis. Wild-type non-drug-treated controls were routinely run in parallel with experimental samples.

### Electrophysiology

For patch-clamp analysis, animals were glued and placed in the recording chamber as described above. ASH cell bodies (identified by GCaMP3 expression) were exposed for whole-cell recordings by partially dissecting the animals as described above, taking care to distinguish ASH neurons and ASI neurons. Dissection quality was validated by ensuring that the dendrite of the exposed ASH had not been severed. Whole cell recordings were performed as previously described (Zahratka et al., 2015). Briefly, we used pressure-polished patch pipettes (Goodman and Lockery, 2000) with 12–30 MΩ resistance containing low Cl^–^ internal solution (15 mM KCl, 115 mM K gluconate, 10 mM HEPES, 5 mM MgCl_2_, 0.25 mM CaCl_2_, 5 mM EGTA, 20 mM sucrose, 5 mM MgATP, 0.25 mM NaGTP, pH 7.20, 315 mOsm). OA was directly applied on the exposed ASH using a perfusion pencil (AutoMate Scientific, Berkeley, CA, United States) as described above and delivered using a syringe pump (KD Scientific, Holliston, MA, United States). 1-oct was delivered as described above and the cells were observed after each 1-oct application for SR101 staining to ensure there had not been inadvertent exposure of the cell soma to the 1-oct solution. Signals were recorded with an Axopatch 200B amplifier (Molecular Devices, Sunnyvale, CA, United States) in current-clamp mode (0 pA injected current, 10 kHz sampling, 2 kHz filtering), digitized with a Digidata 1440A digitizer, and analyzed using pCLAMP10 software (Molecular Devices, Sunnyvale, CA, United States).

### Behavioral assays

Behavioral responses to 1-oct were assayed as previously described (Chao et al., 2004; Harris et al., 2009). L4 animals were picked the night before the assay onto fresh NGM plates seeded with OP50. Plates containing 4 mM 5-HT, 4 mM OA, or 4 mM 5-HT + 4 mM OA were prepared fresh, 2 h before the experiment. 1-oct was diluted to 30% in 100% ethanol (v/v) and was presented to a forward moving animal *via* a glass capillary. For assays in the absence of any monoamine, animals were transferred from their regular stock plate to a food free intermediate plate for 1 min to remove any OP50 and then transferred to a food free assay plate and tested 10 min later. In contrast, for assays in the presence of monoamines, animals were transferred to the respective monoamine containing assay plate after the intermediate plate and tested 30 min later. All reagents were obtained from Fisher Scientific (Waltham, MA, United States) or Sigma-Aldrich (St. Louis, MO, United States).

### Experimental design and statistical analysis

For behavioral experiments, a minimum of 20 young adult hermaphrodites were analyzed for each treatment/mutation. For Ca^2+^ imaging and electrophysiology experiments, minimum of five young adult hermaphrodites were recorded each day, where practical. All experiments were performed between 19°C and 23°C. All reagents were prepared fresh before the experiment. Statistical analysis was performed using unpaired, two-tailed Student’s *t*-tests or one-way ANOVA with Tukey post-test, using GraphPad Prism software (San Diego, CA, United States) with data presented as mean ± SD.

## Results

### Octopamine reduces somal Ca^2+^ transients in ASHs but antagonizes 5-HT modulation of aversive behavior

**FIGURE 1:**
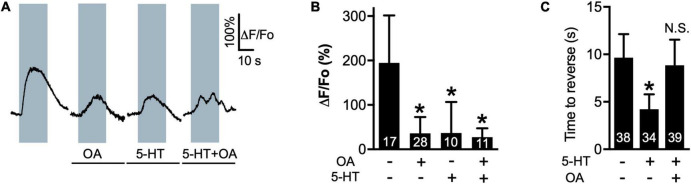
OA reverses 5-HT modulation of 1-oct avoidance behavior but does not reverse 5-HT modulation of somal Ca^2+^ transients in ASH. **(A)** Representative traces of ASH somal Ca^2+^ transients in response to 1-oct exposure in control (far left), OA, 5-HT, and 5-HT + OA treated worms. Gray boxes represent duration of 1-oct exposure. **(B)** Quantification of 5-HT and OA effects on 1-oct-induced ASH somal Ca^2+^ transients. **(C)** OA antagonizes 5-HT potentiation of 1-oct avoidance behavior (30% 1-oct stimulus). *Significantly different from untreated control (*p* < 0.0001, ANOVA). N.S., not significantly different compared with untreated control (*p* > 0.05, ANOVA). ΔF/F_o_, change in fluorescence relative to original fluorescence intensity. Values are mean ± SD. Numbers within bars indicate *n*.

5-HT decreases the amplitude of 1-oct-dependent Ca^2+^ transients in the soma of ASH neurons while increasing depolarization amplitude and potentiating 1-oct-stimulated avoidance behavior, dependent on SER-5/Gα_q_ signaling within ASHs (Harris et al., 2009; Zahratka et al., 2015; Williams et al., 2018b). Like 5-HT, OA (4 mM) also reduces 1-oct-stimulated Ca^2+^ transients in the ASH soma (Figures 1A,B; baseline Ca^2+^ levels were not affected by the monoamines, alone or in combination, Supplementary Figure S1). OA does not affect 30% 1-oct avoidance behavior on its own, but does antagonize 5-HT potentiation, dependent on the cell autonomous effects of the OCTR-1 OA receptor in ASHs (Wragg et al., 2007). We confirmed the OA antagonism of 5-HT potentiation (Figure 1C). Despite reversing the 5-HT effect on behavior, OA does not reverse the effect of 5-HT on 1-oct-dependent Ca^2+^ transients in the ASH soma when worms are co-incubated with both monoamines (Figures 1A,B). These results imply that 5-HT and OA activate different signaling pathways which converge on the optically measurable somal Ca^2+^ transient, and indicate that, in terms of behavior, the OA pathway dominates the 5-HT pathway when both are active simultaneously. These results also reinforce the idea that somal Ca^2+^ transient modulation may not be a reliable predictor of a neuron’s output to the downstream locomotory circuitry.

### Octopamine decreases Ca^2+^ transient amplitudes through OCTR-1 and G_o_ signaling

**FIGURE 2:**
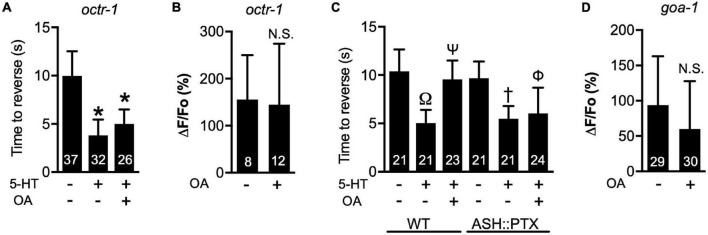
OCTR-1 and GOA-1 are required for OA effects on 1-oct-stimulated avoidance behavior and ASH somal Ca^2+^ signals. **(A)** OA fails to antagonize the 5-HT potentiation of aversive behavior in *octr-1* null mutants. **(B)** OA fails to inhibit ASH somal Ca^2+^ transients in *octr-1* null mutants. **(C)** The ability of OA to reverse 5-HT signaling is evident in wild type animals (left three bars), but absent in ASH::PTX animals (right three bars) (30% 1-oct stimulus). 5-HT signaling is unaffected in ASH::PTX animals. **(D)** OA does not inhibit ASH somal Ca^2+^ transients in *goa-1* null mutants. *Significantly different from untreated control (*p* < 0.0001, ANOVA). N.S., not significantly different compared with untreated control (*p* > 0.05, unpaired *t*-test). ^Ω^Significantly different from untreated wild type (*p* < 0.0001, ANOVA). ^Ψ^Significantly different from 5-HT treated wild type (*p* < 0.0001, ANOVA) but not significantly different from untreated wild type (*p* > 0.05, ANOVA). ^†^Significantly different from untreated ASH::PTX animals (*p* < 0.0001, ANOVA) but not significantly different from 5-HT treated wild type. ^φ^Not significantly different from 5-HT treated ASH::PTX animals (*p* > 0.05, ANOVA) but significantly different from untreated ASH::PTX animals (*p* < 0.0001, ANOVA. Values are mean ± SD. Numbers within bars indicate *n*.

To better understand how OA signaling affects ASH responses to 1-oct, we further investigated the OA intracellular signaling cascade. We investigated *octr-1* null mutants to test the potential role for the OCTR-1 receptor, based on previous behavioral results (Wragg et al., 2007). OCTR-1 is a GPCR in *C. elegans* homologous to the mammalian α2A adrenoceptor (Sellegounder et al., 2018). OCTR-1 binds OA with high affinity and tyramine with lower affinity (Wragg et al., 2007), and activates G_o_ signaling when expressed in *Xenopus* oocytes (Mills et al., 2012). The *octr-1* promoter drives expression in ASHs as well as several other neurons and non-neuronal cells (Wragg et al., 2007). OCTR-1 is involved in innate immunity and the unfolded protein response in *C. elegans* (Liu et al., 2016; Sellegounder et al., 2018), and plays a key role in the OA modulation of 1-oct aversive responses (Wragg et al., 2007; Mills et al., 2012). First, we confirmed that OCTR-1 was essential for OA to counteract 5-HT potentiation of 1-oct avoidance behavior in our hands (Figure 2A, compare to Figure 1C), which had been reported previously (Wragg et al., 2007). We then showed the OA-dependent reduction in 1-oct-stimulated Ca^2+^ transients in the ASH soma was absent in *octr-1* mutants (Figure 2B). These findings suggest that OCTR-1 initiates a signaling cascade which inhibits both ASH somal Ca^2+^ transients and avoidance behavior. OCTR-1 couples to G_o_ in *Xenopus* oocytes (Mills et al., 2012) and G_o_ signaling is required in ASHs for OA signaling based on neuron-specific RNAi experiments (Harris et al., 2010). To provide further evidence for G_o_ signaling involvement, we examined worms expressing PTX under the control of the *osm-10* promoter, which expresses strongly in ASH and weakly in ASI (Hofler and Koelle, 2011). PTX inactivates G_o_ signaling through ADP-ribosylation of Gα_o_ (Gierschik, 1992; Darby and Falkow, 2001). OA inhibition of 5-HT potentiation was defective in these transgenic strains, while 5-HT potentiation itself, and OA inhibition in wild type controls tested in the same experiment, were both normal (Figure 2C), confirming a role for G_o_ signaling downstream of OCTR-1 in OA modulation of aversive behavior. Unfortunately, we could not test OA modulation of ASH somal Ca^2+^ transients in the ASH::PTX transgenic strains because they also expressed a strong GFP marker in ASH which interfered with GCaMP imaging. Instead, we performed Ca^2+^ imaging in ASHs of *goa-1* mutants, which lack Gα_o_ and are therefore defective in G_o_ signaling (Ségalat et al., 1995). OA failed to inhibit 1-oct induced somal ASH Ca^2+^ transients in the *goa-1* background (Figure 2D), demonstrating a role for G_o_ signaling downstream of OCTR-1 in OA modulation of somal Ca^2+^ transient amplitudes as well. Taken together, these results support a model in which OA activates OCTR-1 and G_o_ signaling in ASHs, leading to reduced somal Ca^2+^ transient amplitudes in ASHs and behavioral inhibition.

### Octopamine signaling does not modulate olfactory transduction or L-VGCC activation

**FIGURE 3:**
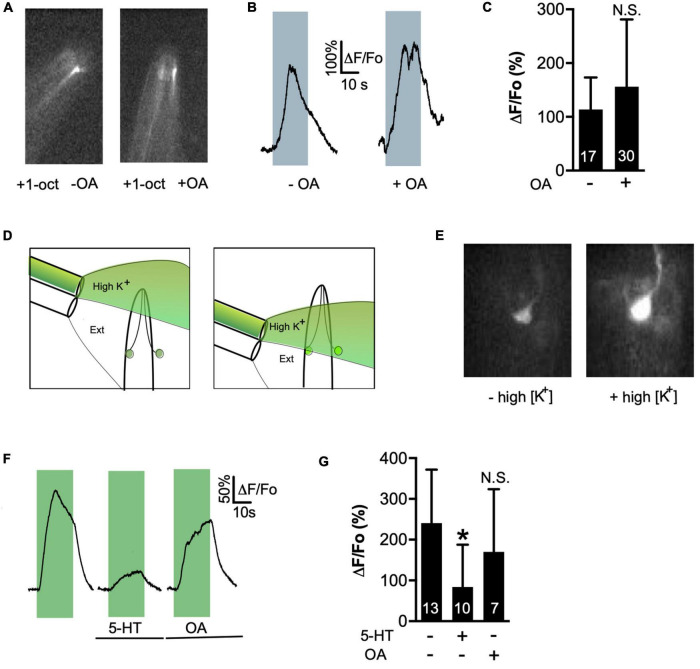
OA does not inhibit primary signal transduction of 1-oct or L-VGCC activity. **(A,B)** Ca^2+^ transients induced by 1-oct in wild-type ASH amphids. Gray boxes in **(B)** represent duration of 1-oct exposure. **(C)** Quantification of amphid Ca^2+^ signals. N.S., not significantly different from untreated counterpart (*p* = 0.1966, unpaired *t*-test). **(D)** Experimental approach for high K^+^ stimulation of ASH neurons, showing the two-barrel puffer used to deliver the stimulus. Puffer is mounted on a motorized drive and can be moved to deliver stimuli at precise intervals (i.e., compare left and right panels). Neuron on right is partially dissected and exposed to bath. **(E)** Images of GCaMP3 fluorescence in a dissected ASH soma before (left) and during (right) exposure to 30 mM K^+^ solution. **(F)** Representative traces of high K^+^-stimulated somal Ca^2+^ transients. Green boxes represent duration of high K^+^ exposure. **(G)** Quantification of 5-HT and OA effects on high K^+^-stimulated somal Ca^2+^ signals. *Significantly different from untreated control (*p* = 0.0196, ANOVA). N.S., not significantly different from untreated control (*p* = 0.4799, ANOVA). Values are mean ± SD. Numbers within bars indicate *n*.

How does OCTR-1/G_o_ signaling modulate ASH responses to 1-oct? Gα_o_ can inhibit olfactory transduction in mammalian olfactory receptor cells (Corey et al., 2021), suggesting that OA could be inhibiting primary sensory transduction at the ASH cilia, at the amphid openings. Ca^2+^ influxes in the ASH cilia are due to activation of OSM-9/OCR-2 TRP channel, downstream of odorant binding to its receptor (Ferkey et al., 2021). Therefore, these influxes can be interpreted as a marker of the efficiency of primary sensory transduction, upstream of any potential effect on cellular excitability (Williams et al., 2018b). 1-oct exposure caused a sharp rise in the Ca^2+^ signal in the amphid region which was not significantly decreased by OA incubation (Figures 3A–C), suggesting that OA did not affect the primary olfactory transduction cascade.

An alternative mechanism for OA to reduce ASH Ca^2+^ transients could be direct inhibition of L-type VGCCs by OA signaling. We previously observed this phenomenon in the ASH soma, where 5-HT signaling through SER-5/Gα_q_ induced the release of Ca^2+^ from intracellular stores, leading to calcineurin (CaN)-dependent dephosphorylation and inactivation of the L-VGCC (Williams et al., 2018b). G_o_ signaling can also directly inhibit L-VGCC activation through Gβγ subunits, which are released upon dissociation of activated heterotrimeric G proteins (Waterman, 2000). To test whether these mechanisms were operating, we depolarized neurons with high K^+^-containing external solution after partially dissecting worms to expose the soma of one ASH neuron to the bath. Exposure to solutions containing 30 mM K^+^ reliably depolarizes neurons leading to activation of L-VGCCs, which allows direct inhibition of L-VGCCs by monoamine signaling to be detected (Williams et al., 2018b). Importantly, this approach bypasses any upstream steps in the neuronal activation pathway, so any effect of monoamine signaling would necessarily be due to modulation of L-VGCC open channel probabilities (Williams et al., 2018b). Switching from normal external solution to high K^+^ external solution led to robust Ca^2+^ transients in the soma of bath-exposed ASH neurons (Figures 3D,E). These transients were strongly inhibited by 5-HT treatment, as previously reported (Williams et al., 2018b), but were not significantly reduced by OA treatment (Figures 3F,G). These results reinforce our conclusion that while 5-HT and OA have similar quantitative effects on somal Ca^2+^ transients in ASH neurons, the underlying signal transduction pathways of the two monoamines are very different.

### Octopamine signaling requires GIRK channels

**FIGURE 4:**
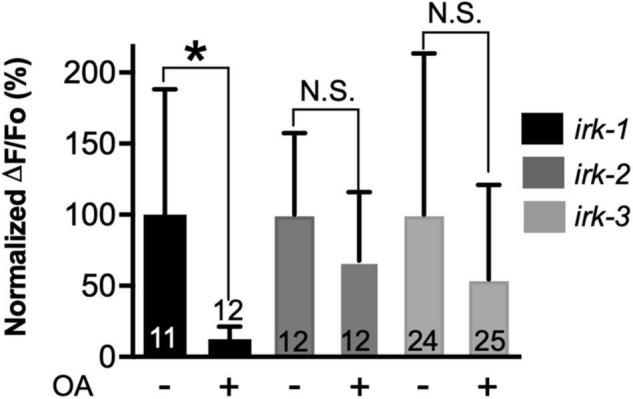
Octopamine inhibition of 1-oct-induced somal Ca^2+^ transients in ASHs requires IRK-2 and IRK-3. *Significantly different from untreated control (*p* < 0.005, unpaired *t*-test). N.S., not significantly different compared with untreated control (*p* > 0.05, unpaired *t*-test). Values are mean ± SD. Numbers within bars indicate *n*.

G_o_ signaling can also hyperpolarize neuronal membranes through the activation of GIRK channels by G_βγ_ subunits (Kano et al., 2019). OCTR-1, heterologously expressed in *Xenopus* oocytes, is capable of signaling in this manner (Mills et al., 2012). The *C. elegans* genome contains three GIRK genes (*irk-1*, *irk-2*, and *irk-3*), which are widely expressed in the nervous system (Emtage et al., 2012; Hobert, 2013; WormBase, 2022). Therefore, we hypothesized that these IRK proteins could play a role downstream of OCTR-1/G_o_. To test the potential involvement of GIRK channels in OA signaling, we performed calcium imaging in the three different GIRK null mutants (*irk-1*, *irk-2*, and *irk-3*). OA did not significantly inhibit ASH somal Ca^2+^ transients in the *irk-2* and *irk-3* null backgrounds but produced normal inhibition in the *irk-1* background (Figure 4). This result indicates that IRK-2 and IRK-3 are required downstream of OCTR-1/G_o_ signaling and suggests that the OA inhibition of ASH somal Ca^2+^ transients is due to reduction of ASH excitability. Behavioral analysis revealed a role for all three GIRK channels in OA modulation of 1-oct avoidance, but the results did not cleanly correspond to the circumscribed role for these channels predicted by our model for OA signaling in ASH (see Discussion), possibly due to the expression of IRK channels in neurons other than ASHs, and the involvement of additional circuits and OA signaling pathways controlling 1-oct aversive responses (Supplementary Figure S2).

### Octopamine inhibits ASH depolarization

**FIGURE 5:**
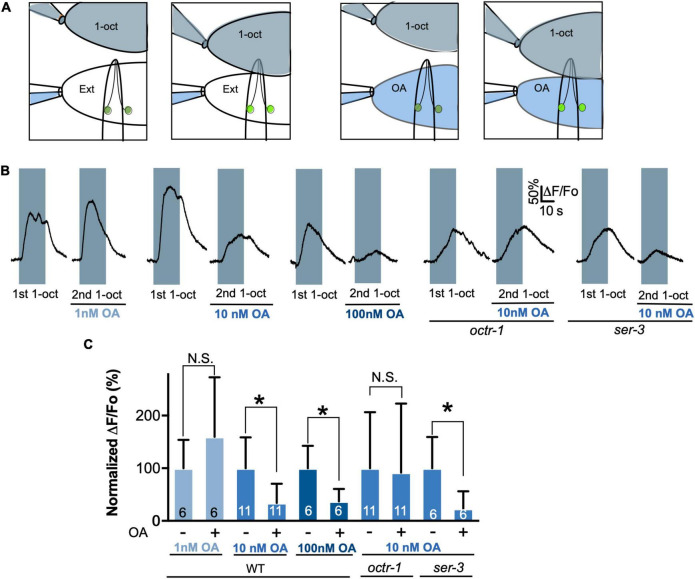
Determination of the physiological concentration range for OA/OCTR-1 signaling. **(A)** Diagram illustrating dual pipette perfusion system. Upper pipette delivers the olfactory stimulus, which is a saturated solution of 1-oct in external solution (shaded gray). Pipette is mounted on a motorized drive, which is moved to deliver stimuli at precise intervals. Lower pipette delivers external solutions, with or without OA, separated from one another by a glass septum that allows for rapid switching. This stream also deflects the 1-oct solution away from the exposed cell body to prevent direct contact of the exposed cell soma with 1-oct. **(B)** Representative traces of 1-oct-stimulated ASH somal Ca^2+^ transients at various OA concentrations in wild type and OA receptor mutants. Gray boxes represent duration of 1-oct exposure. **(C)** Quantification of 1-oct induced ASH somal Ca^2+^ transients. *Significantly different from untreated control (*p* < 0.05, paired *t*-test). N.S., not significantly different compared with untreated control (*p* > 0.05, paired *t*-test). Values are mean ± SD. Numbers within bars indicate *n*.

To determine whether OA reduces ASH excitability, we performed direct electrophysiological recordings on the ASHs. These recordings are performed on partially dissected worms in which an ASH soma is exposed to the bath, taking care not to sever the dendritic process connecting the soma to the amphid opening (Zahratka et al., 2015; Williams et al., 2018b). Dissection and proper placement of flow pipettes generally takes longer than the perdurance of OA signaling when worms are pre-incubated on OA plates, which was the approach used in the above experiments (see section “Materials and Methods”). Therefore, it was necessary to treat ASHs with OA after they had been dissected. Incubation of *C. elegans* with monoamines on plates requires very high concentrations because of low cuticular permeability to polar compounds, which prevents accurate determination of effective internal monoamine concentrations (Williams et al., 2018a). Therefore, it was first necessary to determine a minimal effective concentration of OA for these electrophysiological experiments. Using Ca^2+^ imaging in the exposed ASH soma as a readout, we performed paired recordings on individual neurons before and after exposure to OA at 1, 10, and 100 nM for 60 s (Figure 5A). 1-oct responses were unaltered at 1 nM OA, but significantly decreased at 10 and 100 nM (Figures 5B,C), establishing 10 nM as an appropriate OA concentration, comparable to the minimal effective concentrations observed for 5-HT receptors from *C. elegans* and other species (Adolph and Tuan, 1972; Bunin and Wightman, 1998). Significantly, *octr-1* mutants did not show reduced 1-oct dependent Ca^2+^ transients after 10 nM OA treatment (Figures 5B,C), confirming that OCTR-1 is the relevant receptor for OA inhibition of ASH somal Ca^2+^ transients under these conditions. A second OA receptor, SER-3, also acts in ASHs at higher OA concentrations (Mills et al., 2012), but *ser-3* mutants still showed inhibition of 1-oct Ca^2+^ transients in the presence of 10 nM OA, confirming the central role for OCTR-1 in OA modulation of somal Ca^2+^ transients in ASH.

**FIGURE 6:**
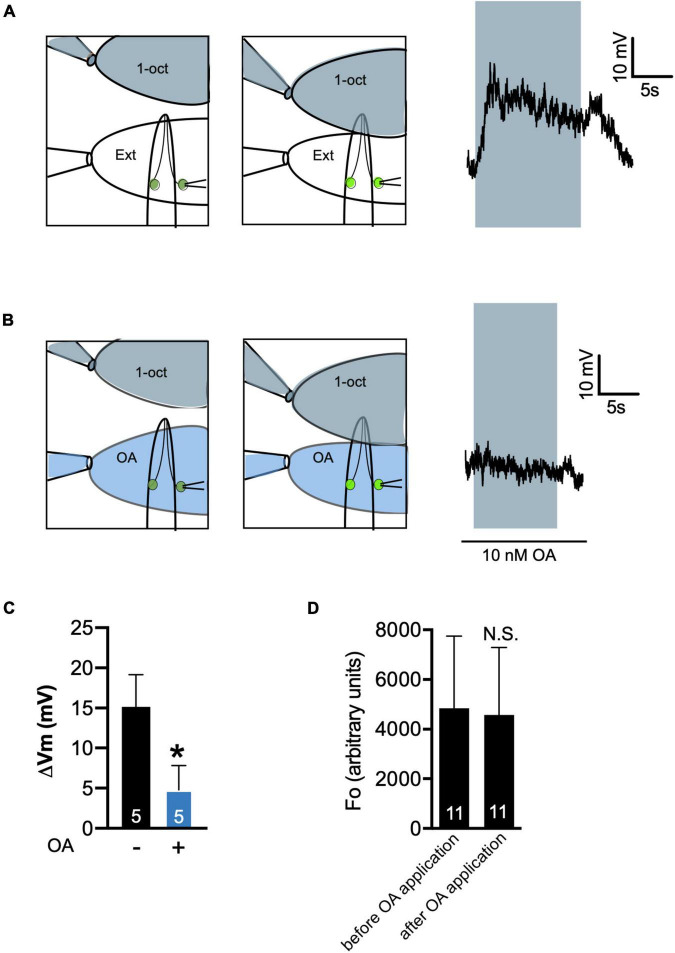
Octopamine inhibits 1-oct-stimulated depolarization in ASH neurons. **(A,B)** Recording protocol (left) and representative trace (right) of 1-oct-stimulated depolarization in ASH in the absence **(A)** and presence **(B)** of 10 nM OA. 1-Oct solution shaded gray, 10 nM OA shaded blue, neuron on right exposed to bath to provide access to the patch pipette. Gray box over trace indicates duration of 1-oct exposure. **(C)** Quantification of OA inhibition of ASH depolarization. ΔV_m_, change in membrane potential. **(D)** OA treatment does not decrease resting Ca^2+^ levels in the soma of ASH neurons. Average somal GCaMP3 fluorescence values in bath-exposed ASH neurons before and after 10 nM OA application. N.S., not significantly different compared with untreated control (*p* > 0.05, paired *t*-test). *Significantly different from untreated control (*p* < 0.005, unpaired *t*-test). Values are mean ± SD. Numbers within bars indicate *n*.

Patch clamp recordings demonstrated that OA treatment reduced the amplitude of 1-oct-induced depolarization in ASHs. In control samples, exposing the tip of the worm’s nose to 2.2 mM 1-oct in external solution (a saturating concentration) resulted in a slowly developing +15 mV depolarization which returned to baseline following 1-oct removal (Figures 6A,C), comparable to previously published results (Zahratka et al., 2015; Williams et al., 2018b). The placement of flow pipettes provided a constant stream of clean external solution to the ASH soma, shielding it from direct 1-oct exposure (Figure 6A), so we can confidently attribute the observed depolarization to olfactory signaling at the ASH sensory ending (Zahratka et al., 2015), not the direct exposure of the ASH soma to 1-oct. Sixty second pre-treatment of the ASH soma with 10 nM OA significantly reduced this 1-oct response (Figures 6B,C). While OA signaling inhibited 1-oct-dependent depolarization, it did not appear to hyperpolarize ASH neurons at rest, since somal Ca^2+^ levels in individual bath-exposed ASHs were not decreased by 10 nM OA application (Figure 6D). This result implies that the membrane potential of unstimulated ASH neurons may be at or near the K^+^ reversal potential. Based on these results, we conclude that OA inhibition of 1-oct-stimulated Ca^2+^ transients in the soma of ASH neurons reflects G_o_-dependent activation of GIRK channels leading to reduced neuronal depolarization, which stands in contrast to 5-HT inhibition of ASH somal Ca^2+^ transients, which reflects Gα_q_-dependent inhibition of L-VGCCs leading to enhanced neuronal depolarization.

## Discussion

Neuromodulators increase or decrease neuronal excitability in a graded manner to regulate neural circuit outputs. Measuring these changes using Ca^2+^ imaging in intact circuits is a critical tool for understanding how neuromodulators affect animal behavior. Therefore, it is essential to correctly interpret quantitative changes in Ca^2+^ transient amplitudes when studying neuromodulator actions at the circuit level. Because neuronal Ca^2+^ transients are the result of the activation of VGCCs, it is frequently assumed that increased Ca^2+^ transient amplitudes indicate increased depolarization amplitudes and vice versa. However, ASH neurons in *C. elegans* exhibit the opposite correlation when modulated by 5-HT, with decreasing somal Ca^2+^ transients indicating increased depolarization. To determine whether this unexpected relationship holds true consistently for this cell type, we investigated how another neuromodulator, OA, affects the ASH neuron. We show that OA utilizes a completely independent signaling pathway, inhibiting ASH somal Ca^2+^ transients and ASH depolarization in parallel. Therefore, quantitative relationships between somal Ca^2+^ signal amplitudes and depolarization amplitudes may be different even within a single cell type.

**FIGURE 7:**
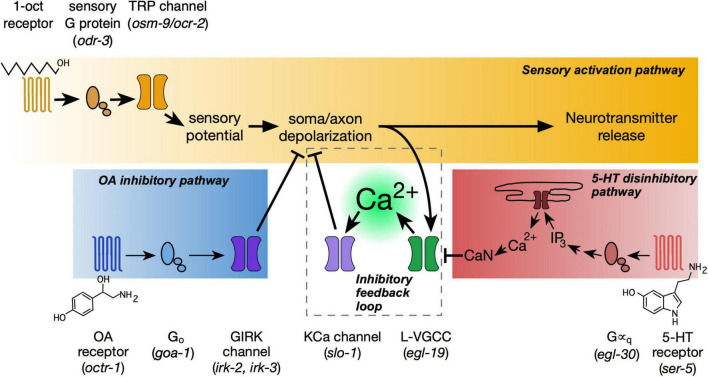
Interacting signaling pathways within the ASH neuron regulate the strength of sensory responses. The sensory activation pathway (gold) depolarizes the neuron, leading to synaptic vesicle release and excitatory signaling to downstream aversive locomotory circuitry. Ca^2+^ entering through L-VGCCs, responsible for optically measurable somal Ca^2+^ transients, provides negative feedback on depolarization (dashed box). 5-HT signaling (red) inhibits this Ca^2+^-mediated inhibition, and therefore disinhibits depolarization. Finally, OA signaling (blue) blocks sensory-dependent depolarization through OCTR-1, G_o_, and GIRK channels. Identity of signaling proteins indicated by text above and below pathways. Corresponding mutants shown in parentheses.

Detailed analysis of ASH signaling reveals three signaling pathways operating in an integrated manner to regulate excitability (Figure 7). The main sensory activation pathway depolarizes the neuron, which leads to synaptic vesicle fusion, the release of neurotransmitters onto postsynaptic targets, and initiation of aversive locomotory responses (Bargmann et al., 1993; Chao et al., 2004; Zahratka et al., 2015). However, this pathway branches to activate L-VGCCs, which produce the optically detectable Ca^2+^ signals in the ASH soma (Mills et al., 2012; Zahratka et al., 2015). This Ca^2+^ influx does not facilitate or amplify depolarization but actually inhibits it, through negative feedback. This interpretation is supported by action of Nemadipine A, a L-VGCC blocker, which demonstrably reduces the Ca^2+^ influx while increasing depolarization amplitude (Williams et al., 2018b). 5-HT signaling impinges on the negative feedback branch of the sensory activation pathway. 5-HT activates Gα_q_ signaling (Harris et al., 2009), leading to release of Ca^2+^ from intracellular stores and the activation of CaN, resulting in dephosphorylation and inhibition of the L-VGCC (Williams et al., 2018b). Thus, for 5-HT, optically measurable Ca^2+^ transients in the ASH soma decrease while ASH depolarization amplitude increases (Zahratka et al., 2015; Williams et al., 2018b). OA signaling impinges on the main sensory activation pathway. Through the OCTR-1 receptor, OA activates G_o_ signaling resulting in GIRK channel activation, presumably through the release of Gβγ subunits, thus inhibiting the initial 1-oct dependent depolarization. Surprisingly, the recently released CeNGEN neuronal gene expression profile dataset did not show *octr-1* or *irk-2* expression in ASH neurons (Taylor et al., 2021; WormBase, 2022). However, expression of several other genes which function in adult ASHs was also not detected (Harris et al., 2009; Aoki et al., 2011; Mills et al., 2012; Chatzigeorgiou et al., 2013; Ezcurra et al., 2016). It is possible that some functionally significant neuronal genes become expressed at higher levels in adults than in L4 larvae (the source of the RNA for the transcriptional profiling), or that low abundance transcripts may still be functionally significant even if below the detection threshold of scRNA-seq. However, we cannot rule possible non-cell autonomous roles for *octr-1* and *irk-2* in modulating ASH responses.

The respective endpoints of 5-HT and OA signaling in our model explain why OA signaling overrides 5-HT signaling, instead of the other way around. When, in the presence of OA, the ASH soma fails to depolarize, L-VGCC activity remains low. There is little somal Ca^2+^ influx and little negative feedback on depolarization. With low L-VGCC activity to begin with, any further reduction due to 5-HT signaling will have a comparatively small impact on overall membrane excitability, and therefore will not overcome the OA effect. Furthermore, open GIRK channels may exert a continuous hyperpolarizing influence, which could compensate for any residual decrease in Ca^2+^-activated K^+^ channel activity due to 5-HT signaling. These insights underscore the value of studying neural circuit dynamics in small, defined model nervous systems such as *C. elegans*. Because of the stereotyped anatomy of *C. elegans*, the pair of ASH neurons may be unambiguously identified from one animal to the next. Therefore, results obtained at different times, with different neuromodulators and different analytical techniques may be cross correlated with high confidence, strongly supporting the interpretation that all three pathways operate together in time and space.

Correctly inferring how neuronal depolarization is modulated based on Ca^2+^ recording is particularly important where membrane potentials undergo graded changes, as opposed to the all-or-none depolarizations seen with action potentials. In *C. elegans* (with a few exceptions) (Mellem et al., 2008; Liu et al., 2018) the vast majority of neuronal communication is believed to rely on graded potentials, because *C. elegans* lacks voltage-gated sodium channels (Bargmann, 1998). *C. elegans* is a powerful model for understanding sensory motor processing and circuit modulation because of its small size and fully characterized neuronal connectivity. Recently, it has also become possible to perform whole-brain recordings in *C. elegans*, in which activity patterns for large numbers of neurons may be recorded simultaneously, with each Ca^2+^ profile assigned to a uniquely identified neuron (Yemini et al., 2021). These powerful tools will significantly advance our knowledge of how intrinsic patterns of neuronal activity are generated, how incoming sensory information impacts those patterns (Kato et al., 2015), and how these circuit dynamics are modulated through monoamine and neuropeptide signaling (Zhang et al., 2005; Harris et al., 2010; Mills et al., 2012; Liu et al., 2022). Comparative genomic, connectomic, and transcriptomic studies have revealed striking parallels in molecular mechanisms and functional organization with mammalian nervous systems, providing a robust framework for applying insights from *C. elegans* to the human brain (Bargmann, 1998; Milo et al., 2002; Varshney et al., 2011; Rengarajan and Hallem, 2016; Arnatkevic̆iūtė et al., 2018). However, graded changes in membrane potential are also important in nervous systems which do utilize action potentials. Dendrites integrate large numbers of synaptic inputs and perform logical computations based on the strength and geometrical distribution of co-incident excitatory and inhibitory postsynaptic potentials (Stuart and Spruston, 2015), studied extensively using Ca^2+^ imaging (Roome and Kuhn, 2018; Ali and Kwan, 2020). Active signaling (i.e., dendritic spiking) is observed, facilitating supra-linear summation of multiple inputs (Stuart and Spruston, 2015). However, passive non-regenerative signaling also plays an important role, facilitating linear and sub-linear summation of inputs, which is critical in primary visual and somatosensory cortices (Longordo et al., 2013; Jia et al., 2014; Stuart and Spruston, 2015). The relationship between dendritic Ca^2+^ signals and depolarization dynamics is clearly complex (Roome and Kuhn, 2018; Ali and Kwan, 2020), and even includes an example, in cortical layer 5 pyramidal neurons, where larger amplitude Ca^2+^ signals in dendritic spines correlate with smaller excitatory postsynaptic potentials at the cell soma dependent on Ca^2+^-activated K^+^ channels (Tazerart et al., 2022), reminiscent of ASH neurons in *C. elegans* in the presence of 5-HT (Williams et al., 2018b). Insights gained from the study of ASH neurons into neuromodulatory mechanisms and, especially, the relationship between Ca^2+^ signaling intensity and membrane excitability may prove to be particularly relevant to dendritic signal processing, given that all of the major signaling proteins involved in modulation of ASHs are found localized to dendrites in mammalian neurons (Halpain et al., 1998; Vardi, 1998; Hartmann et al., 2004; Chen and Johnston, 2005; Segal and Korkotian, 2014; Sekerková et al., 2014; Cha et al., 2019; Xu et al., 2022).

## Data availability statement

The raw data supporting the conclusions of this article will be made available by the authors, without undue reservation.

## Author contributions

AD, PW, and BB contributed to conception of research. AD and BB designed the research, interpreted the results of experiments, prepared the figures, and wrote the manuscript. AD performed the experiments and analyzed the data. All authors contributed to the article and approved the submitted version.

## Conflict of interest

The authors declare that the research was conducted in the absence of any commercial or financial relationships that could be construed as a potential conflict of interest.

## Publisher’s note

All claims expressed in this article are solely those of the authors and do not necessarily represent those of their affiliated organizations, or those of the publisher, the editors and the reviewers. Any product that may be evaluated in this article, or claim that may be made by its manufacturer, is not guaranteed or endorsed by the publisher.
