# Supplementary material for: Reduced Ca2+ transient amplitudes may signify increased or decreased depolarization depending on the neuromodulatory signaling pathway
Source: Front Neurosci. 2022 Jul 22;16:931328. doi: 10.3389/fnins.2022.931328 (PMC9354622; doi:10.3389/fnins.2022.931328)
Supplement: Supplementary file 1 [file Image_1.PDF]

Supplementary material

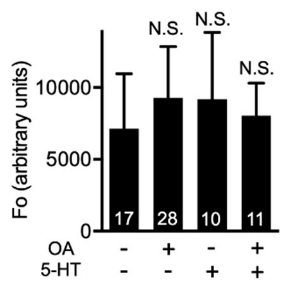

Figure S1: 5-HT and OA treatment do not affect baseline somal Ca^{2+} levels in ASH neurons N.S. Not significantly different compared with untreated control (p>0.05, ANOVA). Values are mean ± SD. Numbers within bars indicate n.

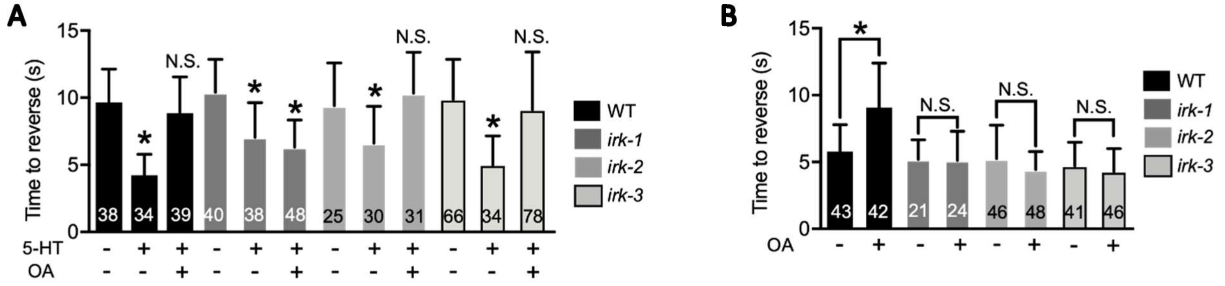

Figure S2: GIRK channel involvement in OA modulation of 1-oct avoidance behavior. For low intensity stimulation (30% 1-oct, A), IRK-1 is necessary for OA behavioral effects, while IRK-2 and IRK-3 are not, which does not agree with our reported results for ASH somal Ca^{2+} transients. For higher intensity stimuli (100% 1-oct, B), all three channels are required, although a different OA receptor (SER-6) is the relevant receptor at this stimulation level. Importantly, IRK-1, IRK-2, and IRK-3 are expressed in other neurons besides ASH, and aversive behavioral responses to 1-oct are reflective of whole-network activity patterns. For example, ASI, AWB, and ADL neurons are known to be involved. These neurons express a different OA receptor (SER-6), which stimulates release of neuropeptides encoded by the nlp-6, 7, 8, and 9 genes (Mills et al., 2012). These peptides slow the 1-oct behavioral responses (from 5s to 10s), mimicking the effects of OA. Loss of IRK channels in ASI, AWB, and ADL could lead to hyperexcitability and abnormal release of nlp-6, 7, 8, and/or 9 peptides, leading to a slowing of 1-oct responses that would mask the expected effects of disrupted OA signaling in ASH. *Significantly different from untreated worms of the same strain (p< 0.05, ANOVA). N.S.: Not significantly different compared with untreated worms of the same strain (p>0.05, ANOVA). Values are mean ± SD. Numbers within bars indicate *n*.
